# Supplementary material for: A novel member of drug/metabolite transporter (DMT) family efflux pump, SA00565, contributes to tetracycline antibiotics resistance in Staphylococcus aureus USA300
Source: Microbiol Spectr. 2024 Apr 23;12(6):e00111-24. doi: 10.1128/spectrum.00111-24 (PMC11237813; doi:10.1128/spectrum.00111-24)
Supplement: Supplemental material — Fig. S1 and S2; Tables S1 and S2. [file spectrum.00111-24-s0001.pdf]

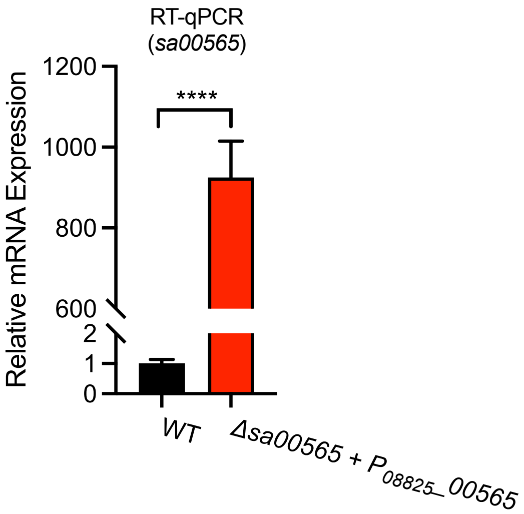

Supplementary Fig. 1. RT-qPCR was employed to analyze the expression level of *sa00565* in both WT and *sa00565* overexpression strains. The fold changes in the expression level of *sa00565* overexpression strains, driven by a robust constitutive promoter, were calculated (****P⩽0.0001, compared to the WT).

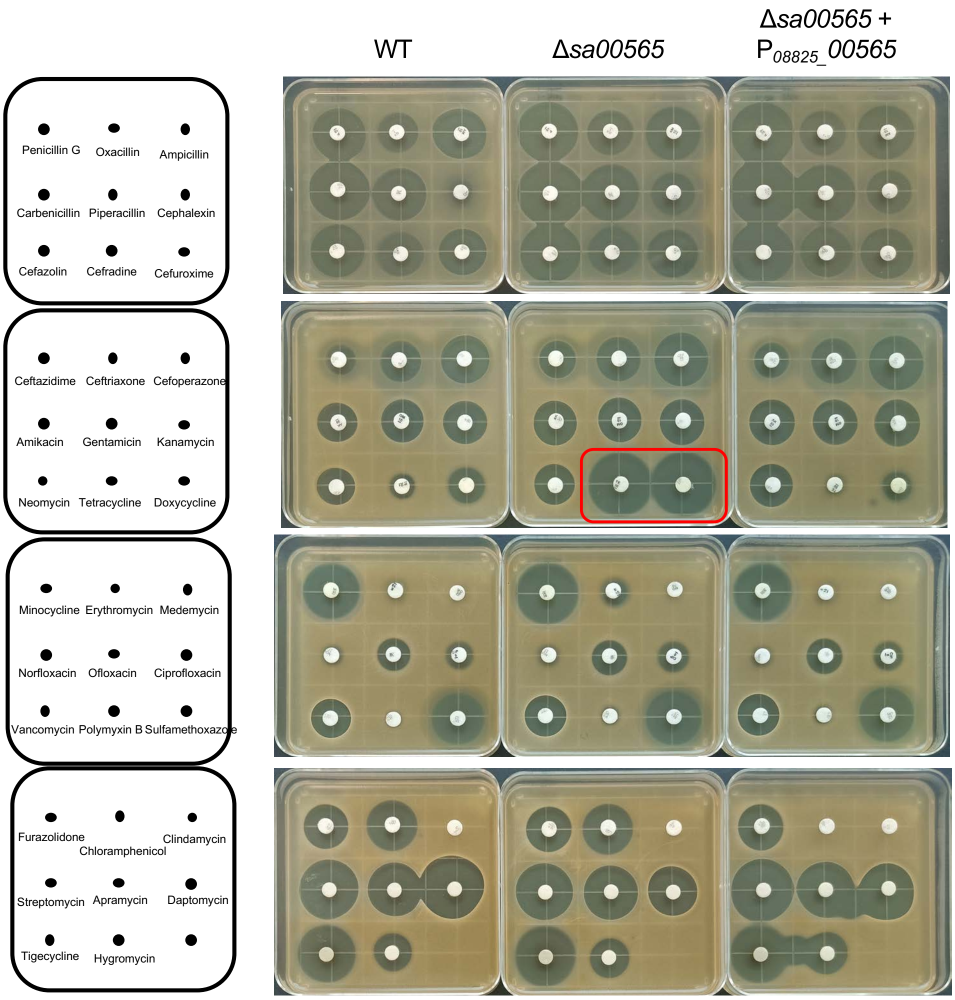

Supplementary Fig. 2. Disk diffusion assay. The susceptibility of *S. aureus* WT, Δ*sa00565*, and *sa00565* overexpression strains against 35 different antibiotics was tested by disk diffusion assay.

Strain Δ*sa00565* exhibited increased susceptibility to antibiotics tetracycline and doxycycline were highlighted with red squares.

Supplementary Table 1. Primers used in this study.

Primer name	Description	Sequence (5' to 3')
QL1195	SAUSA300_RS00565 knockout up F	CACTCATCGCAGTGCAGCGGCTACCGTTT GTCACAAAGACAAG
QL1196	SAUSA300_RS00565 knockout up R	TTATTCCAAGTCCCGCACCTC
QL1197	SAUSA300_RS00565 knockout down F	AGGTGCGGGACTTGGAATAATAATATCA TCACTCCTTTAATTATGTGTTTC
QL1198	SAUSA300_RS00565 knockout down R	CTTGCATGCCTGCAGGTCGACTTATTGCT GGTGCAATCGTT
QL1199	SAUSA300_RS00565 knockout check F	GAAGCTAGACCAGATCCGAT
QL1198	SAUSA300_RS00565 knockout check R	CGTTGTACTGTATCGCCAAC
QL1496	*tet* (L) depletion from pQLV1025 F	CAAAGTTGATCCCTTAACGAT ATTTCACCCTCCAATAATGAGGGC
QL1497	*tet* (L) depletion from pQLV1025 R	ATCGTTAAGGGATCAACTTTGGG
QL1347	SA00565 overexpression F	CATAAAAAAGGAGACATGCATATGAATA AACTTCAAGACACTACTTTTC
QL1348	SA00565 overexpression R	TGGTGATGAGAACCTCTCGAGCATGTATT TTGAAAGAAGTTACTTAAACTAACG
QL1349	RT-qPCR for *sa00565* F	ACGCCTAGTAGAGTGATAATTCCG
QL1350	RT-qPCR for *sa00565* R	GATATACACGGCGCCACTTT
QL0152	RT-qPCR for 16SrRNA F	GCTCGTGTCGTGAGATGTTGG
QL0153	RT-qPCR for 16SrRNA R	TTTCGCTGCCCTTTGTATTGT

Supplementary Table 2. Antimicrobial suscesptibility of *S. aureus* WT and its mutant strains in disk diffusion assay

Antimicrobial agent	Zone diameter of inhibition (mm[a])		
	WT	Δ*sa00565*	Δ*sa00565* + P_{08825}-*00565*
Penicillin G (10 U)	23	24.5	24
Oxacillin (1 μg)	17.5	18	18
Ampicillin (10 μg)	23	23	23
Carbenicillin (100)	26	26.5	26.5
Piperacillin (100 μg)	23.5	24.5	24
Cephalexin (30 μg)	10	12	11.5
Cefazolin (30 μg)	23	24	24
Cefradine (30 μg)	20	21	21.5
Cefuroxime (30 μg)	20.5	21	22
Ceftazidime (30 μg)	12	13.5	13
Ceftriaxone (30 μg)	17	18.5	19
Cefoperazone (75 μg)	20.5	22.5	23
Amikacin (30 μg)	17	18.5	19
Gentamicin (10 μg)	18	18	19
Kanamycin (30 μg)	18	19	19
Neomycin (30 μg)	16	17	18
Tetracycline (30 μg)	10.5	27	N[b]
Doxycycline (30 μg)	15	27.5	12
Minocycline (30 μg)	23.5	26	24.5
Erythromycin (15 μg)	N	N	N
Midecamycin (30 μg)	N	N	N
Norfloxacin (10 μg)	N	N	N
Ofloxacin (5 μg)	13.5	14.5	15
Ciprofloxacin (5 μg)	11.5	13	12.5
Vancomycin (30 μg)	16.5	18	18
Polymyxin B (300 U)	N	N	N
Sulfamethoxazole (30 μg)	22	23.5	23.5
Furazolidone (300 μg)	18.5	19	19
Chloramphenicol (30 μg)	21.5	21	N[c]
Clindamycin (2 μg)	N	N	N
Streptomycin (1 mg)	25	24	26
Apramycin (0.5 mg)	23	23	23.5
Daptomycin (0.1 mg)	26.5	21	23.5
Tigecycline (0.1 mg)	24	25	24.5
Hygromycin (0.5 mg)	16	16	17

[a] Sensitivities were assessed by measuring the diameters of the zones of growth inhibition in three independent assay, and the means ±1 SD were calculated.

[b] No significant inhibition zone was observed.

[c] Increased resistance to chloramphenicol was caused by resistant gene carried by the overexpression plasmid.
